# MiR-125b promotes proliferation and migration of type II endometrial carcinoma cells through targeting TP53INP1 tumor suppressor in vitro and in vivo

**DOI:** 10.1186/1471-2407-11-425

**Published:** 2011-10-05

**Authors:** Feizhou Jiang, Te Liu, Yinyan He, Qin Yan, Xiaoyue Chen, Hui Wang, Xiaoping Wan

**Affiliations:** 1 Department of Obstetrics and Gynecology, Shanghai Jiaotong University Affiliated International Peace Maternity & Child Health Hospital of the China Welfare Institute, Shanghai, China; 2 Department of Obstetrics and Gynecology, Shanghai Jiaotong University Affiliated First People's Hospital, Shanghai, China

## Abstract

**Background:**

Our previous studies have identified that miR-125b was overexpressed in type II endometrial carcinoma (EC) cells compared with type I using microRNAs microarray. Although recent studies have shown the important role of miR-125b in several tumors and overexpression of miR-125b in advanced EC, its function in this disease has not yet been defined. In the present study, we tried to confirm the result of microRNAs microarray and further investigated the functions of miR-125b in EC, and tried to find new downstream targets of miR-125b.

**Methods:**

Differential expression of miR-125b was detected between type II EC cells (KLE, AN3CA) with ER negative and type I EC cells (ishikawa, RL95-2) with ER positive by qRT-PCR and northern blotting. The effects of miR-125b of on proliferation, migration, and target protein expression were evaluated by CCK8 assay, wound healing assay, transwell migration assay, western blotting, and Tumorigenicity assays in nude mice. In addition, luciferase reporter plasmid was constructed to demonstrate the direct target of miR-125b.

**Results:**

MiR-125b was overexpressed in type II EC cells compared with type I. Exogenous miR-125b expression increased proliferation and migration of ishikawa cells and abrogating expression of miR-125b suppressed proliferation, and migration of AN3CA cells in vitro. In addition, in vivo tumor formation assay confirmed that forced miR-125b expression promoted proliferation potential of ishikawa cells, and tumor suppressor gene Tumor Protein 53-Induced Nuclear Protein 1 (TP53INP1) was identified to be the direct target of miR-125b.

**Conclusions:**

TP53INP1 was newly identified to be the direct downstream target of miR-125b. MiR-125b, which was overexpressed in type II EC cells compared with type I, contributes to malignancy of type II EC possibly through down-regulating TP53INP1.

## Background

Endometrial carcinoma (EC) is one of the most common gynecologic malignancies and it is totally classified into two subtypes referred to as type I and type II EC [1]. Type I EC, occurring in ~85% of patients, often displays ER positive. Tumors with this type tend to be well differentiated, of low grade and good prognosis. In contrast, type II, consisting mostly of serous and clear cell carcinoma, typically arises in atrophic endometrium via a mechanism unrelated to estrogen exposure. This type is usually ER negative, and poorly differentiated, of high grade and poor prognosis. Although type II ECs account for approximately 15% of cases, they are responsible for about 50% of all relapses [2]. Great progress was made in the therapies of type II EC, but its 5-year survival rate is still less than 15%. Molecular mechanisms explaining the development and progression of type II EC are still unknown.

MicroRNAs are ~22 nucleotide (nt) non-coding RNAs that function as sequence-specific regulators of gene expression through translational repression and/or transcript cleavage [3-6]. Since the roles and functions of endogenously expressed microRNAs were first described in *C. elegans *in 1993 [7], more and more studies have shown that microRNAs play key roles in cellular processes of differentiation, proliferation, apoptosis and metabolic homeostasis. MicroRNAs profiles have shown that there was much microRNAs expression variation across the different subtypes and stages of carcinogenesis, with data indicating that they may play vital roles in the initiation and progression of human malignancies [8,9]. In cancer tissues, microRNAs appear to be dysregulated such that those with tumor-suppressor activity are abrogated, while those that are overexpressed may function as oncogenes promoting proliferation, migration and invasion, and repressing apoptosis respectively. Recent data showed that overexpression or lack of expression of certain specific microRNAs were correlated to metastatic and aggressive clinical phenotypes [10-12]. It was demonstrated that miR-125b was dysregulated in many tumors such as prostate cancer [13], leukemia [14,15] liver cancer [16], and oligodendroglial tumors [17]. In addition, miR-125b has been demonstrated to mediate the proliferative effects through down-regulating p53 [18], pro-apoptotic Bcl-2 antagonist killer 1 (Bak1) [19], and Bcl-2 modifying factor (Bmf) [20]. It has also been proved to be one of etiologic factors of leukemia [21]. Taken together, these observations suggest that miR-125b may play a vital role in the initiation and progression of cancers.

In our previous work, we chose well-differentiated EC cells (ishikawa, RL95-2) with ER positive and metastatic EC cells (KLE, AN3CA) with ER negative as models of type I and type II EC, respectively. Using microRNAs microarray, we found that miR-125b was significantly overexpressed in ER-negative cells, especially in AN3CA cells, when compared with ER-positive cells. Although recent studies have shown the important role of miR-125b in several tumors and overexpression of miR-125b in advanced ECs [22], its function in this disease has not yet been defined. The findings promoted us to hypothesize that miR-125b may contribute to malignancy of type II EC. In the present study, we confirmed the result of microRNAs microarray, and further investigated the functions of miR-125b in EC and found new downstream targets of miR-125b.

## Methods

### Reagents

Four synthetic, chemically modified short single- or double-stranded RNA oligonucleotides: miR-125b mimics (miR-125bm), miR-125b mimics negative control (miR-125bm NC), miR-125b inhibitors (miR-125bi) and miR-125b inhibitors negative control (miR-125bi NC) were synthesized from Shanghai GenePharma Co., Ltd. Commercial miR-125b expression plasmid and control vector were purchased from Shanghai GeneChem Co., Ltd.

### Cell culture and transfection

Ishikawa, RL95-2, KLE, and AN3CA cells were obtained from American Type Culture Collection (ATCC, Manassas, Va) and were maintained DMEM/F12 media (11030; Gibco, Auckland, NZ) supplemented with 10% FBS (S1810; Biowest, Nuaillé, France), 100 units/ml penicillin, and 100 μg/ml streptomycin. All cells were grown in sterile conditions at 37°C in a humidified atmosphere of 5% CO_2 _and 95% air. Before the experiment, the ER expressions of these cells were confirmed by immunocytochemistry. Cells were seeded in 6-well plates at 70-80% confluence and grown overnight before transfection. Transfection of AN3CA cells (or ishikawa cells) with miR-125bi (or miR-125bm) or counterpart negative control using the lipfectamine2000 transfection reagent (Invitrogen, Carlsbad, CA, USA) according to the manufacturer's instructions. The total experiments were classified into three groups, which were miR-125bm (or miR-125bi) group, miR-125bm NC (or miR-125bi NC) group, and untransfected group.

### RNA isolation and qRT- PCR

Total RNA was isolated from cultured cells with Tri-reagent (TR118; Molecular Research Center, Cincinnati, OH, USA) according to manufacturer's instructions. QRT-PCR analyses for microRNAs were performed using TaqMan microRNA assays. In brief, all reagents and primers were obtained from Applied Biosystems. U6 snRNA served as an endogenous control for normalization. Reverse transcriptase reactions and real-time PCR were performed according to the manufacturer's protocols. CDNA was made from 50 ng total RNA from each sample using the microRNA TaqMan Reverse Transcription Kit and miR-125b specific reverse transcription primer. QRT-PCR reactions were performed in triplicate on a RealPlex4 real-time PCR detection system from Eppendorf Co. LTD (Germany), with cycle threshold values determined using the manufacturer's software. Relative expression of miR-125b, relative to U6 snRNA, was calculated using the 2^-delta delta CT ^method.

### Northern blotting analysis

All steps of Northern blotting were performed as previously described [20]. For all groups, 20 μg of good quality total RNA was subjected to electrophoresis on a 15% Urea-PAGE gel, and transferred to a Hybond N+ nylonmembrane (Amersham, Freiburg, Germany). After being UV-cross-linked and baked at 50°C for 30 min, the membrane was prehybridized at 42°C for 4 h and then hybridized with the miR-125b anti-sense StarFire probe, 5'-TCACAAGTTAGGGTCTCAGGGA-3' (IDT, Coralville, IA), to detect the 22-nt miRNA-125b fragments according to the instruction of the manufacturer. After washing, membranes were exposed for 20 ~ 40 h to Kodak XAR-5 films (Sigma-Aldrich). As a positive control, all membranes were hybridized with a human U6 snRNA probe, 5'-GCAGGGGCCATGCTAATCTTCTCTGTATCG-3'. Exposure times for the U6 control probe varied from 15-30 min.

### SiRNA targeting

To inhibit expression of endogenous TP53INP1, we designed and prepared HPLC- purified TP53INP1 siRNA (sense: 5'-TCAGCCTCTGGAACAT-3'; anti-sense: 5'-ATGTTCCAGAGGCTGA-3') and, a scrambled siRNA with no homology to any known human mRNA was used as negative control (sense: 5'-CTGTTAAAAATCCAGG-3'; anti-sense: 5'-CCTGGATTTTTAACAG-3') according to the sequence of the TP53INP1 gene. SiRNA oligonucleotide duplexes were synthesized by Genephama Biotech (Shanghai, China).

### Cell proliferation assay

Cells at 12 h post-transfection were seeded in 96-well plates at 2500 cells/well. After that, cell proliferation was evaluated using the CCK8 (C0038, Beyotime Inst Biotech, China) according to manufacturer's instructions. Briefly, 10 μl of CCK8 solution was added to the culture medium, and incubated for additional 3 h. The absorbance was determined at 450 nm wavelength with a reference wavelength of 630 nm.

### Wound healing assay

Cells (6 × 106 per well) at 12 h post-transfection were seeded in six-well plates and allowed to adhere for 24 h. Confluent monolayer cells were scratched by a 200 μl pipette tip and then washed three times with 1 × PBS to clear cell debris and suspension cells. Fresh serum-free medium was added, and the cells were allowed to close the wound for 48 h. Photographs were taken at 0, 24, and 48 h at the same position of the wound.

### Transwell migration assay

Cells (2 × 10^5^) were resuspended in 200 μl of serum-free medium and seeded on the top chamber of the 8 μm pore, 6.5 mm polycarbonate transwell filters (Corning). The full medium (600 μl) containing 10% FBS was added to the bottom chamber. The cells were allowed to migrate for 24 h at 37°C in a humidified incubator with 5% CO_2_. The cells attached to the lower surface of membrane were fixed in 4% paraformaldehyde at room temperature for 30 min and stained with 4,6-diamidino-2-phenylindole (DAPI) (C1002, Beyotime Inst Biotech, China), and the number of cells on the lower surface of the filters was counted under the microscope. A total of 5 fields were counted for each transwell filter.

### Western blotting analysis

Total proteins extracts of each group cells were resolved by 10% SDS-PAGE and transferred on PVDF (Millipore) membranes. After blocking, the PVDF membranes were washed 4 times for 15 min with TBST at room temperature and incubated with primary antibody (rabbit anti-human TP53INP1 polyclonal antibody; 1:1000, ab9775, Abcam). Following extensive washing, membranes were incubated with secondary peroxidase-linked goat anti-rabbit IgG (1:1000, sc2030, Santa Cruz) for 1 h. After washing 4 times for 15 min with TBST at room temperature once more, the immunoreactivity was visualized by enhanced chemiluminescence (ECL kit, Pierce Biotechnology), and membranes were exposed to Kodak XAR-5 films (Sigma-Aldrich).

### Construction of reporter plasmids and luciferase assay

To construct a reporter plasmid containing the 3'UTR of TP53INP1, 480 bp DNA fragment of partial 3'UTR of TP53INP1 containing the wild-type 3'UTR of TP53INP1 or mutant 3'UTR of TP53INP1 was chemically synthesized and cloned into the pMIR-REPORT luciferase vector (Ambion) downstream of the luciferase gene and named pMIR-TP53INP1-3'UTR-WT and pMIR-TP53INP1-3'UTR-MT, respectively. The nucleotide sequences of the constructed plasmids were confirmed by DNA sequencing analysis. All steps of luciferase reporter assay were according to the previously described [13,23]. NIH-3T3 cells(1 × 10^5^) were seeded into 24-well plates, and each was transfected with 0.5 μg of either pMIR-TP53INP1-3'UTR-WT vector or pMIR-TP53INP1-3'UTR-MT vector containing Firefly luciferase, together with 0.2 μg of the pRL-TK vector (Promega) containing Renilla luciferase and 50nM miR-125bm, miR-125bm NC or none using the Lipofectamine2000 transfection reagent according to the manufacturer's protocol. After 48 h transfection, luciferase activity was measured using the dual luciferase reporter assay system (Promega). The results were expressed as relative luciferase activity (Firely LUC/Renilla LUC).

### Tumorigenicity assays in nude mice

All experimental protocols were approved by the Ethic Committee for Animal Experimentation of Shanghai Jiaotong University. A total of 5 × 10^6 ^cells suspended in 100 μl 1× PBS were injected into the bilateral hind leg subcutaneous tissue of four-week old female BALB/C athymic nude mice. Three groups of mice (n = 12) were tested. MiR-125b plasmid group was injected with ishikawa cells transfected with miR-125b expression plasmid. Control vector group was injected with ishikawa cells transfected with negative control plasmid. During a five-week follow-up period, the sizes of tumors were measured weekly. Mice were sacrificed at 42 days post-injection. Tumors were excised and measured. The short and long diameters of the tumors were measured using a caliper and tumor volumes (cm^3^) were calculated by using the following standard formula: tumor volumes (cm^3^) = (the longest diameter) × ( the shortest diameter)^2 ^× 0.5.

### Immunohistochemistry

Tissues were fixed in 4% paraformaldehyde and embedded in paraffin blocks. Sections (4 μm) were used for immunohistochemical examination. Ki67 protein expression of tumor tissues were detected using standard avidin-biotin immunohistochemical techniques with use of an anti-ki67 antibody (1:100, lab vision, Fremont, USA) according to the manufacturer's instructions. The labeled cell nuclei in tumor sections were regarded as positive. Proliferative tumor cells were counted in 10 randomly different microscopic fields respectively. The proliferative index was calculated as follows: (number of positive tumor cells/number of total tumor cells) × 100%.

### Statistical analysis

Each experiment was performed as least three times, and data are shown as the mean ± SD where applicable, and differences were evaluated using one-way ANOVA for 3-group comparisons and t tests for 2-group comparisons. All statistical analyses were performed using SPSS 13.0 software package. The probability of P < 0.05 was considered to be statistically significant.

## Results

### Differential expression of miR-125b between type I and type II EC cells

**Figure 1:**
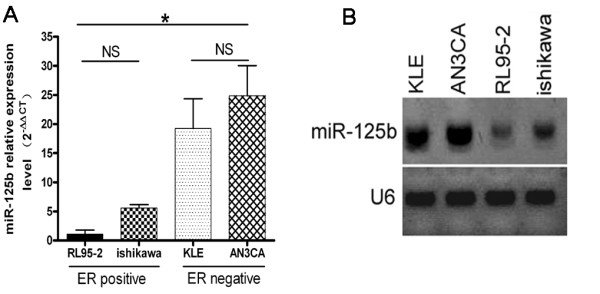
**Expression of miR-125b were assessed in EC cells by qRT-PCR and Northern blotting**. A. Differential expression of miR-125b between type I and type II EC cells. Bars show mean ± SD. All experiments were repeated three times. *P < 0.01 vs. ishikawa, KLE, AN3CA. B. The results of Northern blotting show that the hybridized signal of the mature miR-125b in EC cells with ER negative was stronger than that in EC cells with ER positive.

In our previous work, we found that miR-125b was significantly overexpressed in type II EC cells (KLE and AN3CA) with ER negative compared with type I EC cells (RL95-2 and ishikawa) with ER positive using microRNA microarray (unpublished data). To validate microarray findings, TaqMan based qRT-PCR analysis of differentially expressed miR-125b was performed. In agreement with the microarray results, there was a significant difference of miR-125b expression between ER-negative and ER-positive cells (P < 0.01). As shown in Figure 1A, the expression of miR-125b in KLE cells was 17.36 fold (p = 0.000) and 3.46 fold (p = 0.002) that of RL95-2 and ishikawa, respectively; the expression of miR-125b in AN3CA cells was 22.39 fold (p = 0.000) and 4.46 fold (P = 0.000) that of RL95-2 and ishikawa, respectively; the expression of miR-125b in ishikawa cells was 5.03 fold ( p = 0.173) that of RL95-2. There was a slight increase in expression of miR-125b in AN3CA cells compared with KLE but with no statistical significance. In addition, northern blotting revealed that miR-125b signal in EC cells with ER negative was stronger than in that with ER positive (Figure 1B).

### MiR-125b promoted proliferation of EC cells

**Figure 2:**
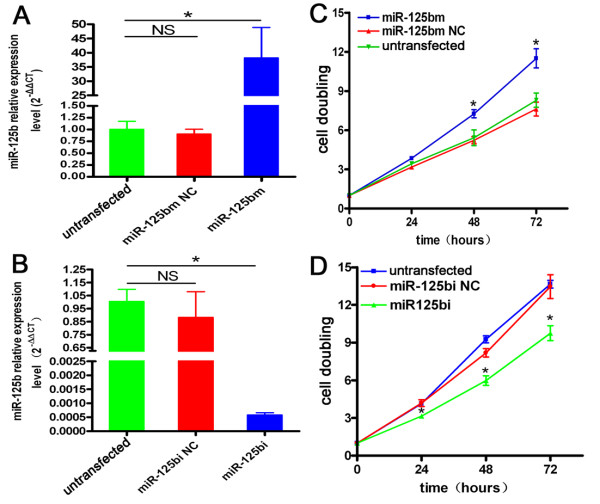
**Analysis of transfection efficiency and cell proliferation after differential treatment**. A. Transfection efficiency of ishikawa cells was measured at 72 h after differential transfecton by qRT-PCR. Bars show mean ± SD. *P < 0.01 vs. miR-125bm NC, untransfected. B. Transfection efficiency of AN3CA cells was measured at 72 h after differential transfecton using qRT-PCR. Bars show mean ± SD. *P < 0.01 vs. miR-125bi NC, untransfected. C. CCK8 analysis of the growth of three groups of ishikawa cells. *P < 0.05 vs. miR-125b NC, untransfected. D. CCK8 analysis of the growth of three groups of AN3CA cells. *P < 0.05 vs. miR-125b NC, untransfected.

To investigate the biological effect of miR-125b, cell proliferation was measured after differential transfection. Transfection efficiency was detected at 72 h post-transfection (Figure 2A and 2B). As shown in Figure 2C, treatment with miR-125bm significantly stimulated the growth of ishikawa cells compared with miR-125bm NC and untransfected (Figure 2C, p < 0.05). MiR-125bi, but not the miR-125bi NC significantly inhibited the growth of AN3CA cells compared with untransfected group (Figure 2D, p < 0.05).

### MiR-125b enhanced the mobility of EC cells

**Figure 3:**
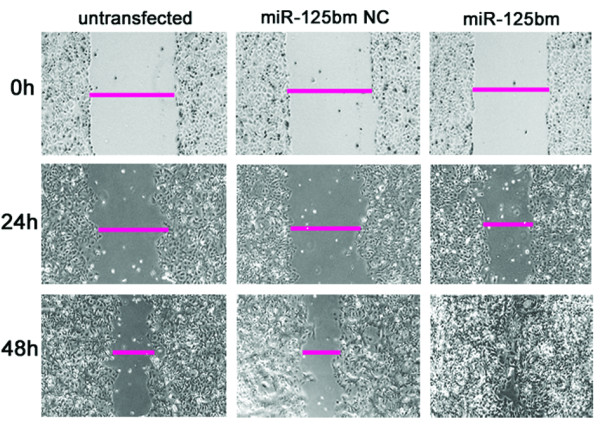
**Effect of miR-125b on cell migration of AN3CA cells after differential treatment in vitro wound healing assay**. Ishikawa cells were seeded in 6-well plates and wounding on the next day. Photographs were taken at hour 0, 24, and 48 h, respectively, after the wound was made. Ishikawa cells transfected with miR-125b, but not miR-125bm NC and untransfected group, closed the wound at 48 h after incubation.

To explore the roles of miR-125b in the regulation of cell mobility, we examined the migration ability of ishikawa and AN3CA cells after different transfection treatment by using wound healing assay and transwell migration assay, respectively. For wound healing assay, ishikawa cells transfected with miR-125bm exhibited an obvious increase in migration rate as compared to miR-125bm NC or untransfected group; and ishikawa cells transfected with miR-125bm, but not miR-125bm NC, closed the wound at 48 h after incubation (Figure 3). AN3CA cells without transfection or transfected with miR-125bi NC nearly closed the wound at 48 h after incubation, whereas AN3CA cells transfected with miR-125bi were unable to close the wound at the same time point (additional file 1).

**Figure 4:**
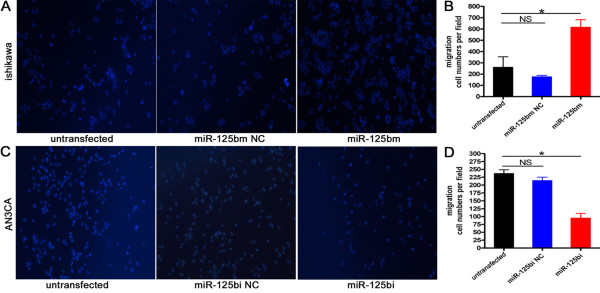
**MiR-125b enhanced the mobility ability of EC cells using transwell migration assay**. A. ishkawa cells were seeded in transwell filter. Migrated cells on the lower surface of the transwell filter were stained and counted after 24 h. Photographs were taken at 24 h post-migration (magnification, 100×). B. The numbers of migrated cells of ishikawa. Bars show mean ± SD. All experiments were repeated three times. *P < 0.01 vs. miR-125bm NC, untransfected. C. AN3CA cells seeded in transwell filter. Migrated cells on the lower surface of the transwell filter were stained and counted after 24 h. photographs were taken at 24 h post-migration (magnification, 100×). D. The numbers of migrated cells of AN3CA. Bars show mean ± SD. All experiments were repeated three times. *P < 0.01 vs. miR-125bi NC, untransfected.

In addition to wound healing assay, transwell migration assay was also performed to confirm the increase in motility of EC cells with overexpressed miR-125b. Consistent with the results in wound healing, migration was significantly promoted in all three miR-125b overexpressed EC cells (ishikawa transfected with miR-125bm, AN3CA without treatment or transfected with miR-125bi NC) (Figure 4A~D). The number of migrated cells of ishikawa cells transfected with miR-125bm was increased nearly 3.5 (or 2.5) fold as compared with miR-125bm NC (or blank) (Figure 4A~B). The number of migrated cells of AN3CA cells transfected with miR-125bi was reduced about 2.5 fold as compared with miR-125bi NC or blank (Figure 4C~D).

These results exhibit a functional role for miR-125b in mediating migration in EC cells and suggest a mechanism by which overexpression of miR-125b may contribute to metastasis of type II EC.

### MiR-125b negatively regulated the expression of TP53INP1

**Figure 5:**
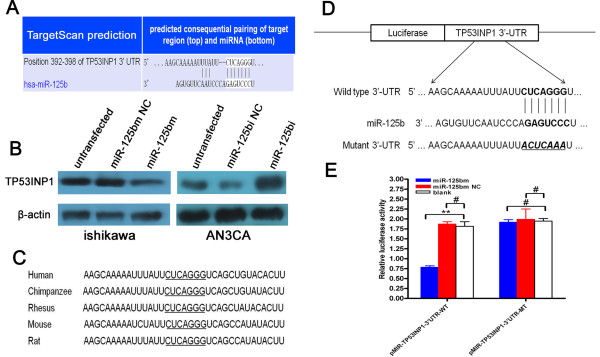
**MiR-125b negatively regulated TP53INP1 protein levels in EC cells**. A. Putative binding sites of miR-125b in the TP53INP1 3'UTR (white sequences) predicted by TargetScan. B. TP53INP1 protein levels were measured in EC cells at 72 h post-transfection by western blot assays. C. Potential binding site of miR-125b on TP53INP1 3'UTR in different species. The seed sequence is underlined. D. Sketch of the construction of pMIR-TP53INP1-3'UTR-WT or pMIR-TP53INP1-3'UTR-MT vectors. The mutant binding site is underlined and italicized. E. MiR-125bm down-regulated luciferase activities controlled by wild-type TP53INP1 3'UTR, but did not affect luciferase activity controlled by mutant TP53INP1 3'UTR (white sequences were mutated). The results are means of three independent experiments ± SD. (**P < 0.01 vs. blank; #P > 0.05 vs. blank).

To search for potential targets of miR-125b that may influence proliferation, migration ability of cells, TargetScan, Pictar-Vert, and Microrna.Org were employed for this purpose. TP53INP1 was found among potential targets of miR-125b combinational predicted by the three softwares. The 3'UTR of TP53INP1 carries a binding site for miR-125b (Figure 5A), suggesting that TP53INP1 mRNA might be a direct target of miR-125b. We therefore evaluated the effect of miR-125b on the expression of TP53INP1 protein in ishikawa (or AN3CA) cells transfected with miR-125bm (or miR-125bi). Transfection with miR-125bm led to nearly 65% down-regulation of TP53INP1 in ishikawa cells compared with miR-125bm NC or untransfected group, whereas treatment with miR-125bi induced 167% increase in the TP53INP1 in AN3CA cells, respectively compared with miR-125bi NC (Figure 5B).

TargetScan analysis indicated that TP53INP1 contains one miR-125b binding site on its 3'UTR, and the sequence of the binding site is highly conserved across different species (chimpanzee, Rhesus, Mouse, Rat, and human) (Figure 5C). To verify that the putative miR-125b binding site in the 3'UTR of TP53INP1 is responsible for regulation by miR-125b, we constructed vectors containing wild-type or mutant 3'UTR of TP53INP1 directly fused to the downstream of the Firefly luciferase gene (Figure 5D). The wild-type or mutant vector was cotransfected into NIH-3T3 cells with miR-125bm, miR-125bm NC or none. The transfection efficiency was normalized by cotransfection with Renilla reporter vector. As shown in Figure 5E, miR-125b significantly decreased the relative luciferase activity of wild-type TP53INP1 3'UTR (more than 60%), whereas the reduction of the luciferase activity with mutant TP53INP1 3'UTR was not as sharp as that observed in the wild-type counterpart, suggesting that miR-125b could directly bind to the 3'UTR of TP53INP1. Taken together, these findings indicate that TP53INP1 is a direct downstream target for miR-125b in EC cells.

### The inhibition of the proliferation and migration of type II EC cells by miR-125bi could be significantly attenuated by the repressing of TP53INP1

**Figure 6:**
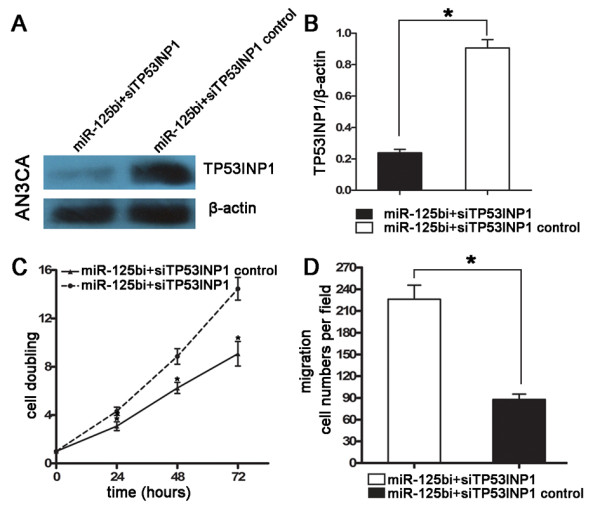
**Repressing TP53INP1 could significantly attenuate the inhibitory effect of miR-125bi on type II EC cell proliferation and migration**. A~B. The expression level of TP53INP1 protein was detected by western blot at 48 h posttransfection and normalized to that of β-actin. The expression level of TP53INP1 protein was significantly lower in cells cotransfected with miR-125bi plus siTP53INP1 as compared to the cells cotransfected with the same inhibitors plus siTP53INP1 control (*P < 0.01). C. Cell proliferation was evaluated by CCK8 analysis. The proliferative capacity of AN3CA cells cotransfected with miR-125bi plus siTP53INP1 was significantly higher than that of cells cotransfected with miR-125bi plus siTP53INP1 control (*P < 0.05). D. Cell migration ability of AN3CA was determined by transwell migration analysis. The number of migrated cells of AN3CA cells cotransfected with miR-125bi plus siTP53INP1 was increased about 2.5 fold as compared that of cells cotransfected with miR-125bi plus siTP53INP1 control (*P < 0.01).

To address whether the above-observed phenotype is indeed due to the suppression of TP53INP1 and not from the targeting of other cellular genes by miR-125b, a rescue experiment was performed. We cotransfected AN3CA cells with miR-125bi plus siTP53INP1 (TP53INP1 siRNA) or with the same inhibitors plus siTP53INP1 control. At 48 h posttransfection, western blot revealed that the expression level of TP53INP1 protein in cells cotransfected with miR-125bi plus siTP53INP1 was significantly lower than that in cells cotransfected with miR-125bi plus siTP53INP1 control (Figure 6A~B, P < 0.01). Indeed, AN3CA cells with stably repressed TP53INP1 were enhanced in both proliferation and migration ability by using CCK8 and transwell migration analysis respectively (Figure 6C, P < 0.05 and Figure 6D, P < 0.01). These results imply that repressing TP53INP1 expression could significantly attenuate the inhibitory effect of miR-125bi on cell proliferation and migration, suggesting that the miR-125b promoted the proliferation and migration of AN3CA cells through targeting TP53INP1 signal pathway.

### Oncogenic role of miR-125b was further demonstrated in vivo tumor xenograft model

**Figure 7:**
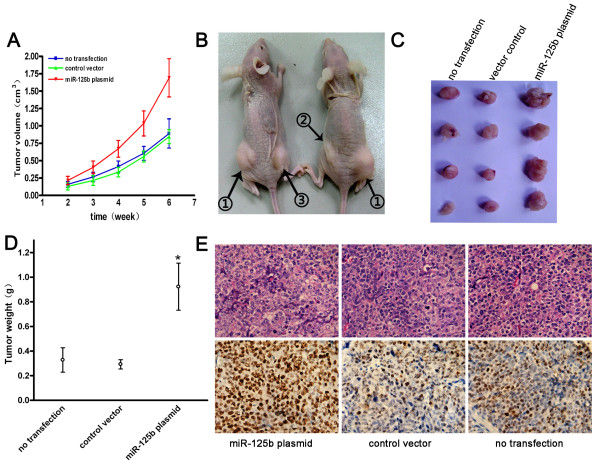
**Tumorigenicity assay in nude mice**. A. Tumor growth curve in nude mice. After tumor cells were injected subcutanecously into the bilateral hind leg of nude mice, the short and long diameters of the tumors were measured weekly and tumor volumes (cm3) were calculated. B. the nude mice with tumor formations. ① indicates the nude mouse injected with ishikawa cells transfected with miR-125b plasmid. ② indicates the nude mouse injected with ishikawa cells transfected with control vector. ③ indicates the nude mouse injected with ishikawa cells with no treatment. C. Photograph of tumors derived from miR-125b plasmid, control vector, or untransfected ishikawa cells in nude mice. D. Weights of tumors. *P < 0.01 as compared with either no transfection group or control vector group. E. Representative HE staining histopathologic image of tumor tissues in mice. (upper panel). ki67 expression of tumors was detected by immunohistochemical techniques (lower panel).

To further determine the role of miR-125b in the progression of EC, we did in vivo animal experiments using ishikawa cells. Tumor formations were observed subcutaneously in all nude mice at 2 weeks after injection. During a five-week follow-up period, it was observed that the tumor volumes were increasing (Figure 7A). At six weeks, the size and weights of tumors were substantially larger in miR-125b plasmid group than those in no transfection group and control vector group, respectively (Figure 7B~D). Furthermore, tumor tissues were embedded in paraffin and then stained with hematoxylin and eosin (H&E) for histology examination (Figure 7E, upper panel). To observe the proliferation ability of ishikawa cells, we performed immunohistochemical staining of ki67, which was expressed as proliferation index. Ki67 protein expression occurred in the nuclei of the tumor cells (Figure 7E, lower panel). The proliferation indexes were (77.40 + 9.29) %, (34.00 +5.61) %, and (35.60 +7.40) %, in miR-125b plasmid group, control vector group, and no transfection group, respectively. Compared with no transfection group and control vector group, miR-125b plasmid group had higher proliferation indexes in xenograft tumor models (p < 0.01). In addition, staining intensity of ki67 in miR-125b plasmid group was much stronger than other groups.

## Discussion

Overexpression of miR-125b has been observed in many cancers such as prostate cancer [13], pancreatic cancer [24], and oligodendroglial tumors [17]. Furthermore, miR-125b plays a vital role in promoting the development of precursor B-cell acute lymphoblastic leukemia and prostate cancer [13,25]. These reports suggested that miR-125b might be associated with tumorigenesis of other types of human tumors. Consistent with these published findings, our results support the concept that miR-125b may act as an oncogene in type II EC. One recent publication was about a comprehensive analysis of the miRNA profile of surgically staged early and advanced endometrial cancer using archival primary ECs tissue samples, and found that expression of miR-125b-1 and miR-125b-2 in stages III and IV ECs was 3.655 and 3.89 fold of stage I endometrioid ECs, respectively[22]. Stages III and IV ECs mostly consist of type II endometrial carcinomas including papillary serous carcinoma and clear cell carcinomas. Previous profiles of microRNAs were totally obtained from microRNAs microarray of archived EC tissue samples, and there was a disadvantage that the findings might be interfered with mesenchymal cells and other non EC components. To solve the problem, we are first, to our knowledge, to choose well-differentiated EC cells with ER positive and metastatic EC cells with ER negative as models of type I and type II EC, respectively. Using microRNAs microarray, we found that miR-125b was significantly overexpressed in type II EC cells with ER negative. Moreover, qRT-PCR was further performed to demonstrate the results of microRNAs microarray. Besides, in our preresearches, we detected endogenous TP53INP1 expression on the archival paraffin-embedded EC specimens of between type I (endometrioid) and type II (papillary serous) patients (each 10 cases) by immunohistochemistry and found that TP53INP1 was expressed in 8/10 type I EC and 1/10 type II EC (unpublished data). Meanwhile, we selected significantly up-regulated miRNAs (n = 6, miR-125b, miR-196b, miR-625, miR-196a*, miR-29a, and miR-140-5P) in our microRNAs microarray (unpublished data) and validated their expression in type I and type II (each 10 cases) EC samples by qRT-PCR. We found that endogenous miR-125b expression was the most significantly up-regulated in type II EC samples compared with type I (additional file 2). These results suggested that the expression level of miR-125b was inversely to endogenous TP53INP1 expression in EC I vs. EC II primary tissues, although the number of the samples is too small to reach a solid conclusion. Our findings were consistent with a previous observation that miR-125b was overexpressed in stages III and IV ECs compared with stage I [22]. MiR-125b is dysregulated in various human cancers but its underlying mechanisms of action are poorly understood.

In current study, exogenous miR-125b expression stimulated the growth and mobility of ishikawa cells, whereas ablation of miR-125b inhibited the growth and mobility of AN3CA cells in vitro. In addition, in vivo xenograft experiment confirmed that forced miR-125b expression promoted proliferation potential of EC cells. We next asked how miR-125b might function inside cells accounting for the effect of miR-125b on biological behavior of EC cells. Using computational search, 148 potential targets of miR-125b were combinational predicted by TargetScan, Pictar-Vert, and Microrna.Org (additional file 3). Among 148 candidates, 4 are potential tumor suppressors including Bmf, adenomatous polyposis coli (APC), START domain containing 13 (STARD13), and TP53INP1. Bmf and STARD13 mRNA was not detectable in these EC cells. Aberrant APC expression, involving WNT signal pathway, was scarcely reported in publication about type II EC. Therefore, we focused on TP53INP1 involving p53 signal pathways.

The main pathway alterations in type II ECs involve p53 which, as a multifunctional transcription factor, plays a central role in cell cycle regulation and genetic stability. Over 90% type II ECs harbor mutant p53 with loss of function [26,27]. TP53INP1 is a proapoptotic stress-induced p53 target gene. It is a tumor suppressor gene with a known role in cellular homeostasis through its antiproliferative and proapoptotic activities via both p53-dependent and p53-independent means [28,29]. However, in the absence of wild type p53 in type II ECs, it may be more important for TP53INP1 to maintain cell homeostasis. There is still no research about TP53INP1 in ECs. However findings in recent years have shown a significant reduction or loss of TP53INP1 expression during the development of cancers of the breast [30], stomach [31], and pancreas [32]. Besides TP53INP1 protein negativity was significantly associated with aggressive pathological phenotypes of gastric cancer; and TP53INP1-positive rate decreased with the progression of gastric cancer [31]. These suggest that TP53INP1 play an important role in suppression of tumor progression [31-34]. MicroRNAs are estimated to regulate the expression of over 30% genes and their functions [35,36], suggesting a possibility that some specific microRNAs might directly regulate TP53INP1. With respect to miRNAs which may regulate the expression of TP53INP1, several studies had reported that TP53INP1 was repressed by miR-155 in pancreatic cancer [32] and miR-130b in hepatocelluar cancer [37], respectively. However, miR-155 and 130b were not among differential expression miRNAs profiles in our studies. In present study, we newly identified miR-125b as the direct regulator of TP53INP1. MiR-125b can directly repress the TP53INP1 protein expression through its binding to the binding sites in 3'UTR of human TP53INP1 gene, thereby negatively regulating TP53INP1 functions. In addition to TP53INP1, three recent studies reported miR-125b can directly target p53 [18], Bak1 [19], and Bmf [20] to enhance the initiation and progression of tumor. Thus, miR-125b might have many other targets that remain to be discovered in future.

The findings here show a regulatory interaction between miR-125b and the tumor suppressor protein, TP53INP1. The results agree with observations elsewhere that aberrant loss of TP53INP1 correlates with the proliferation and migration, but not metastasis [37,38]. This study not only will allow for better understanding of mechanisms which contributes to malignancy of type II ECs, but also will provided sight into the gradual importance of more effective cancer therapies against this disease.

Although some of the results presented here are novel, this study has some limitations. Firstly, the number of the cells line is too small to reach a solid conclusion. In addition, it is required further to evaluate the expression of TP53INP1 and miR-125b simultaneously in large type II EC tissue samples.

## Conclusions

In present studies, we found that miR-125b was overexpressed cultured type II EC cells compared with type I. exogenous miR-125b expression stimulated the growth and mobility of ishikawa cells, whereas ablation of miR-125b inhibited the growth and mobility of AN3CA cells in vitro. In addition, in vivo tumor formation assay confirmed that forced miR-125b expression promoted proliferation potential of EC cells. Tumor suppressor gene TP53INP1 was newly identified to be the direct downstream target of miR-125b. MiR-125b contributes to malignancy of type II EC possibly through down-regulating TP53INP1.

## Abbreviations

EC: endometrial carcinoma; ER: estrogen receptor; qRT-PCR: quantitative real-time reverse transcription polymerase chain reaction assays; CCK8: Cell Counting Kit 8; FBS: fetal bovine serum; PBS: phosphate-buffered saline; 3'UTR: 3'untranslated region; SDS: sodium dodecylsulfate; PAGE: polyacrylamide gel electrophoresis; TBST: Tris-buffered saline with Tween-20; NS: not significant

## Competing interests

The authors declare that they have no competing interests.

## Authors' contributions

FJ carried out the experimental studies and drafted and completed the manuscript. TL participated in the design of the study. YH and QY performed the tedious proofreading. HW participated in tumor pathological characterization. XC completed previous microRNAs microarray. XW conceived of the study and performed the statistical analysis. All authors read and approved the final manuscript.

## Pre-publication history

The pre-publication history for this paper can be accessed here:

http://www.biomedcentral.com/1471-2407/11/425/prepub

## Supplementary Material

Additional file 1 **Effect of miR-125b on cell migration of AN3CA cells after differential treatment in vitro wound healing assay**. AN3CA cells were seeded in 6-well plates and wounding on the next day. Photographs were taken at hour 0, 24, and 48 h, respectively, after the wound was made. AN3CA cells without transfection and transfected with miR-125bi NC nearly closed the wound at 48 h after incubation, whereas AN3CA cells transfected with miR-125bi were unable to close the wound at the same time point. Click here for file

Additional file 2 **Endogenous miR-125b expression was the most significantly up-regulated in type II EC samples compared with type I**. QRT-PCR was performed to validate the expression of endogenous miRNAs (n = 6, miR-125b, miR-196b, miR-625, miR-196a*, miR-29a, and miR-140-5P), which were significantly up-regulated in our microRNAs microarray, in type I (endometrioid) and type II (papillary serous) EC samples. The results showed that endogenous miR-125b expression was the most significantly up-regulated in type II EC samples compared with type I. Click here for file

Additional file 3 **Shown are 148 potential targets of miR-125b which were combinational predicted by TargetScan, Pictar-Vert, and Microrna.Org**. Click here for file
