# Supplementary figures and images for: MiR-125b promotes proliferation and migration of type II endometrial carcinoma cells through targeting TP53INP1 tumor suppressor in vitro and in vivo
Source: BMC Cancer. 2011 Oct 5;11:425. doi: 10.1186/1471-2407-11-425 (PMC3210504; doi:10.1186/1471-2407-11-425)

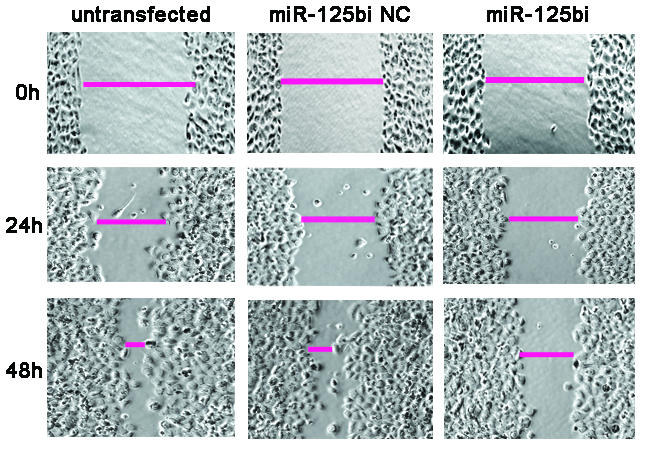

Supplement: Additional file 1 — Effect of miR-125b on cell migration of AN3CA cells after differential treatment in vitro wound healing assay. AN3CA cells were seeded in 6-well plates and wounding on the next day. Photographs were taken at hour 0, 24, and 48 h, respectively, after the wound was made. AN3CA cells without transfection and transfected with miR-125bi NC nearly closed the wound at 48 h after incubation, whereas AN3CA cells transfected with miR-125bi were unable to close the wound at the same time point. [file 1471-2407-11-425-S1.TIFF]

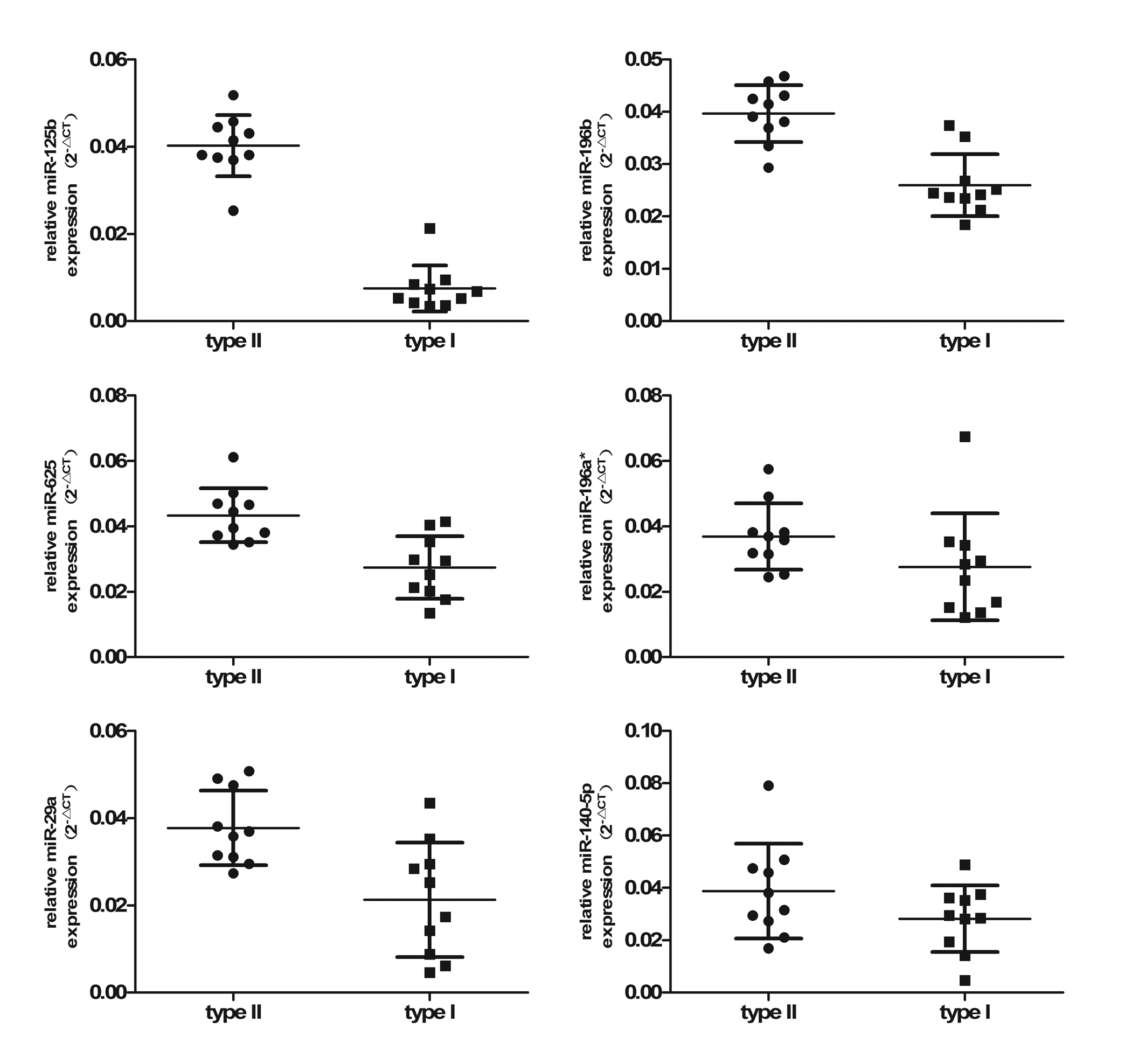

Supplement: Additional file 2 — Endogenous miR-125b expression was the most significantly up-regulated in type II EC samples compared with type I. QRT-PCR was performed to validate the expression of endogenous miRNAs (n = 6, miR-125b, miR-196b, miR-625, miR-196a*, miR-29a, and miR-140-5P), which were significantly up-regulated in our microRNAs microarray, in type I (endometrioid) and type II (papillary serous) EC samples. The results showed that endogenous miR-125b expression was the most significantly up-regulated in type II EC samples compared with type I. [file 1471-2407-11-425-S2.TIFF]
