# Supplementary material for: MiR-125b promotes proliferation and migration of type II endometrial carcinoma cells through targeting TP53INP1 tumor suppressor in vitro and in vivo
Source: BMC Cancer. 2011 Oct 5;11:425. doi: 10.1186/1471-2407-11-425 (PMC3210504; doi:10.1186/1471-2407-11-425)
Supplement: Additional file 3 — Shown are 148 potential targets of miR-125b which were combinational predicted by TargetScan, Pictar-Vert, and Microrna.Org. [file 1471-2407-11-425-S3.DOC]

**Additional file 3 Gene targets of miR-125b combinational predicted by TargetScan, Pictar-Vert and Microrna.Org.**

Homo sapiens START domain containing 13 (STARD13), transcript variant alpha /beta/ gamma, mRNA

Homo sapiens fucosyltransferase 4 (alpha (1,3) fucosyltransferase, myeloid-specific) (FUT4), mRNA

Homo sapiens immediate early response 3 interacting protein 1 (IER3IP1), mRNA.

Homo sapiens interferon regulatory factor 4 (IRF4), mRNA.

Homo sapiens vacuolar protein sorting 4B (yeast) (VPS4B), mRNA.

Homo sapiens lactamase, beta (LACTB), nuclear gene encoding mitochondrial protein, transcript variant 1, mRNA.

Homo sapiens nucleoporin 210kDa (NUP210), mRNA.

Homo sapiens BRCA1 associated protein-1 (ubiquitin carboxy-terminal hydrolase) (BAP1), mRNA.

Homo sapiens glutamyl aminopeptidase (aminopeptidase A) (ENPEP), mRNA.

Homo sapiens AT rich interactive domain 3B (BRIGHT- like) (ARID3B), mRNA.

Homo sapiens tumor necrosis factor (ligand) superfamily, member 4 (tax-transcriptionally activated glycoprotein 1, 34kDa) (TNFSF4), mRNA.

Homo sapiens phosphatidylcholine transfer protein (PCTP), mRNA.

Homo sapiens podocalyxin-like (PODXL), mRNA.

Homo sapiens solute carrier family 39 (zinc transporter), member 9 (SLC39A9), mRNA.

Homo sapiens SLIT and NTRK-like family, member 6 (SLITRK6), mRNA.

Homo sapiens cyclin J (CCNJ), mRNA.

Homo sapiens protein tyrosine phosphatase, non-receptor type 18 (brain-derived) (PTPN18), mRNA.

Homo sapiens oxysterol binding protein-like 9 (OSBPL9), transcript variant 1/2/3/4/5/6/7, mRNA.

Homo sapiens phosphoribosyl pyrophosphate amidotransferase (PPAT), mRNA.

Homo sapiens cingulin (CGN), mRNA.

Homo sapiens ER degradation enhancer, mannosidase alpha-like 1 (EDEM1), mRNA.

Homo sapiens myeloid cell leukemia sequence 1 (BCL2-related) (MCL1), transcript variant 1/2, mRNA.

Homo sapiens sema domain, immunoglobulin domain (Ig), transmembrane domain (TM) and short cytoplasmic domain, (semaphorin) 4D (SEMA4D), mRNA.

Homo sapiens solute carrier family 4, sodium bicarbonate transporter-like, member 10 (SLC4A10), mRNA.

Homo sapiens glypican 4 (GPC4), mRNA.

Homo sapiens sialidase 1 (lysosomal sialidase) (NEU1), mRNA.

Homo sapiens trans-golgi network protein 2 (TGOLN2), mRNA.

Homo sapiens solute carrier family 7 (cationic amino acid transporter, y+ system), member 1 (SLC7A1), mRNA.

Homo sapiens Rap guanine nucleotide exchange factor (GEF) 5 (RAPGEF5), mRNA.

Homo sapiens myelin transcription factor 1 (MYT1), mRNA.

Homo sapiens Bcl2 modifying factor (BMF), transcript variant1/2/ 3/4, mRNA.

Homo sapiens ninein (GSK3B interacting protein) (NIN), transcript variant 2, mRNA.

Homo sapiens somatostatin receptor 3 (SSTR3), mRNA.

Homo sapiens likely ortholog of mouse limb-bud and heart gene (LBH), mRNA.

Homo sapiens mannan-binding lectin serine protease 1 (C4/C2 activating component of Ra-reactive factor) (MASP1), transcript variant 1, mRNA.

Homo sapiens estrogen-related receptor alpha (ESRRA), mRNA.

Homo sapiens zinc finger, FYVE domain containing 1 (ZFYVE1), transcript variant 1/2, mRNA.

Homo sapiens ubiquitin-conjugating enzyme E2R 2 (UBE2R2), mRNA.

Homo sapiens ATPase, Class V, type 10D (ATP10D), mRNA.

Homo sapiens sterile alpha motif domain containing 10 (SAMD10), mRNA.

Homo sapiens regulatory factor X-associated ankyrin-containing protein (RFXANK), transcript variant 1/2, mRNA.

Homo sapiens TBC1 (tre-2/USP6, BUB2, cdc16) domain family, member 1 (TBC1D1), mRNA.

Homo sapiens thymine-DNA glycosylase (TDG), mRNA.

Homo sapiens ankyrin repeat and SOCS box-containing 13 (ASB13), mRNA.

Homo sapiens abhydrolase domain containing 3 (ABHD3), mRNA.

Homo sapiens mitogen-activated protein kinase kinase kinase 10 (MAP3K10), mRNA.

Homo sapiens PR domain containing 1, with ZNF domain (PRDM1), transcript variant 1/2, mRNA.

Homo sapiens scavenger receptor class B, member 1 (SCARB1), mRNA.

Homo sapiens cleavage and polyadenylation specific factor 6, 68kDa (CPSF6), mRNA.

Homo sapiens solute carrier family 35, member A4 (SLC35A4), mRNA.

Homo sapiens solute carrier family 17 (sodium-dependent inorganic phosphate cotransporter), member 7 (SLC17A7), mRNA.

Homo sapiens chromosome 9 open reading frame 86 (C9orf86), mRNA.

Homo sapiens UDP-N-acetyl-alpha-D-galactosamine:polypeptide N-acetylgalactosaminyltransferase 14 (GalNAc-T14) (GALNT14), mRNA.

Homo sapiens sulfotransferase family 4A, member 1 (SULT4A1), transcript variant 1/ 2, mRNA.

Homo sapiens potassium voltage-gated channel, delayed-rectifier, subfamily S, member 3 (KCNS3), mRNA.

Homo sapiens synovial apoptosis inhibitor 1, synoviolin (SYVN1), transcript variant 1/2, mRNA.

Homo sapiens ets variant gene 6 (TEL oncogene) (ETV6), mRNA.

Homo sapiens PHD finger protein 15 (PHF15), mRNA.

Homo sapiens PI-3-kinase-related kinase SMG-1 (SMG1), transcript variant 1/ 2, mRNA.

Homo sapiens solute carrier family 4, sodium bicarbonate cotransporter, member 4 (SLC4A4), mRNA.

Homo sapiens abhydrolase domain containing 6 (ABHD6), mRNA.

Homo sapiens mitogen-activated protein kinase kinase kinase 11 (MAP3K11), mRNA.

Homo sapiens mannosidase, alpha, class 1B, member 1 (MAN1B1), mRNA.

Homo sapiens eukaryotic translation initiation factor 2C, 2 (EIF2C2), mRNA.

Homo sapiens alanyl (membrane) aminopeptidase (aminopeptidase N, aminopeptidase M, microsomal aminopeptidase, CD13, p150) (ANPEP), mRNA.

Homo sapiens suppressor of variegation 3-9 homolog 1 (Drosophila) (SUV39H1), mRNA.

Homo sapiens ribosomal protein S6 kinase, 90kDa, polypeptide 1 (RPS6KA1), transcript variant 1, mRNA.

Homo sapiens CDC42 small effector 1 (CDC42SE1), mRNA.

Homo sapiens tumor protein p53 inducible nuclear protein 1 (TP53INP1), mRNA.

Homo sapiens KIAA0174 gene product (KIAA0174), mRNA.

Homo sapiens tumor necrosis factor, alpha-induced protein 3 (TNFAIP3), mRNA.

Homo sapiens likely ortholog of kinesin light chain 2 (KLC2), mRNA.

Homo sapiens ankyrin repeat and BTB (POZ) domain containing 1 (ABTB1), transcript variant 1/2/3, mRNA.

Homo sapiens mitofusin 1 (MFN1), nuclear gene encoding mitochondrial protein, transcript variant 1/2, mRNA.

Homo sapiens SH3-domain binding protein 4 (SH3BP4), mRNA.

Homo sapiens sema domain, immunoglobulin domain (Ig), transmembrane domain (TM) and short cytoplasmic domain, (semaphorin) 4C (SEMA4C), mRNA.

Homo sapiens proprotein convertase subtilisin/kexin type 7 (PCSK7), mRNA.

Homo sapiens myeloid leukemia factor 2 (MLF2), mRNA.

Homo sapiens ATP-binding cassette, sub-family C (CFTR/MRP), member 5 (ABCC5), mRNA.

Homo sapiens mitogen-activated protein kinase kinase 7 (MAP2K7), mRNA.

Homo sapiens RNA binding motif protein 7 (RBM7), mRNA.

Homo sapiens golgi autoantigen, golgin subfamily a, 5 (GOLGA5), mRNA.

Homo sapiens sema domain, immunoglobulin domain (Ig), transmembrane domain (TM) and short cytoplasmic domain, (semaphorin) 4B (SEMA4B), transcript variant 1/2, mRNA.

Homo sapiens MAM domain containing 2 (MAMDC2), mRNA.

Homo sapiens sphingosine-1-phosphate lyase 1 (SGPL1), mRNA.

Homo sapiens immediate early response 2 (IER2), mRNA.

Homo sapiens UDP-N-acetyl-alpha-D-galactosamine:polypeptide N-acetylgalactosaminyltransferase 7 (GalNAc-T7) (GALNT7), mRNA.

Homo sapiens leucyl/cystinyl aminopeptidase (LNPEP), mRNA.

Homo sapiens phosphatidylinositol 4-kinase type-II beta (PI4K2B), mRNA.

Homo sapiens suppressor of variegation 4-20 homolog 2 (Drosophila) (SUV420H2), mRNA.

Homo sapiens mitochondrial tumor suppressor 1 (MTUS1), nuclear gene encoding mitochondrial protein, transcript variant 1/2/4/5, mRNA.

Homo sapiens adenomatosis polyposis coli (APC), mRNA.

Homo sapiens DAZ associated protein 2 (DAZAP2), mRNA.

Homo sapiens Rap guanine nucleotide exchange factor (GEF)-like 1 (RAPGEFL1), mRNA.

Homo sapiens hypothetical protein FLJ36031 (FLJ36031), mRNA.

Homo sapiens tafazzin (cardiomyopathy, dilated 3A (X-linked); endocardial fibroelastosis 2; Barth syndrome) (TAZ), transcript variant 1/2/3/4/5, mRNA.

Homo sapiens KIAA1598 (KIAA1598), mRNA.

Homo sapiens protein phosphatase 2 (formerly 2A), catalytic subunit, alpha isoform (PPP2CA), mRNA.

Homo sapiens mitogen-activated protein kinase 14 (MAPK14), transcript variant 1/2/4, mRNA.

Homo sapiens ubiquitin specific protease 38 (USP38), mRNA.

Homo sapiens coronin, actin binding protein, 2A (CORO2A), transcript variant 1/2, mRNA.

Homo sapiens thioredoxin reductase 1 (TXNRD1), transcript variant 1/2/4/5, mRNA.

Homo sapiens ectonucleoside triphosphate diphosphohydrolase 4 (ENTPD4), mRNA.

Homo sapiens mannose-6-phosphate receptor (cation dependent) (M6PR), mRNA.

Homo sapiens SERTA domain containing 3 (SERTAD3), transcript variant 1/2, mRNA.

Homo sapiens DEAD (Asp-Glu-Ala-Asp) box polypeptide 42 (DDX42), transcript variant 1/2, mRNA.

Homo sapiens cyclin M1 (CNNM1), mRNA.

Homo sapiens musashi homolog 1 (Drosophila) (MSI1), mRNA.

Homo sapiens ATP-binding cassette, sub-family C (CFTR/MRP), member 4 (ABCC4), mRNA.

Homo sapiens potassium voltage-gated channel, subfamily H (eag-related), member 7 (KCNH7), transcript variant 1, mRNA.

Homo sapiens T-box 4 (TBX4), mRNA.

Homo sapiens ELOVL family member 6, elongation of long chain fatty acids (FEN1/Elo2, SUR4/Elo3-like, yeast) (ELOVL6), mRNA.

Homo sapiens EMI domain containing 1 (EMID1), mRNA.

Homo sapiens flotillin 2 (FLOT2), mRNA.

Homo sapiens glutaminase (GLS), mRNA.

Homo sapiens RAB3D, member RAS oncogene family (RAB3D), mRNA.

Homo sapiens signal transducer and activator of transcription 3 (acute-phase response factor) (STAT3), transcript variant 1/2/3, mRNA.

Homo sapiens ras homolog gene family, member T2 (RHOT2), mRNA.

Homo sapiens trichorhinophalangeal syndrome I (TRPS1), mRNA.

Homo sapiens estrogen-related receptor gamma (ESRRG), transcript variant 1/2/3, mRNA.

Homo sapiens D4, zinc and double PHD fingers family 2 (DPF2), mRNA.

Homo sapiens nuclear receptor co-repressor 2 (NCOR2), mRNA.

Homo sapiens a disintegrin and metalloproteinase domain 9 (meltrin gamma) (ADAM9), transcript variant 1, mRNA.

Homo sapiens eukaryotic translation initiation factor 4E binding protein 1 (EIF4EBP1), mRNA.

Homo sapiens platelet-activating factor acetylhydrolase, isoform Ib, alpha subunit 45kDa (PAFAH1B1), mRNA.

Homo sapiens zinc finger protein 76 (expressed in testis) (ZNF76), mRNA.

Homo sapiens protein phosphatase 2, regulatory subunit B (B56), gamma isoform (PPP2R5C), transcript variant 1/2, mRNA.

Homo sapiens bromodomain adjacent to zinc finger domain, 2A (BAZ2A), mRNA.

Homo sapiens pleiomorphic adenoma gene-like 1 (PLAGL1), transcript variant 1/2, mRNA.

Homo sapiens chromosome X open reading frame 23 (CXorf23), mRNA.

Homo sapiens integrin, alpha 9 (ITGA9), mRNA.

Homo sapiens lysyl oxidase-like 1 (LOXL1), mRNA.

Homo sapiens Dicer1, Dcr-1 homolog (Drosophila) (DICER1), transcript variant 1/2, mRNA.

Homo sapiens ubiquitin domain containing 1 (UBTD1), mRNA.

Homo sapiens eukaryotic translation initiation factor 5A2 (EIF5A2), mRNA.

Homo sapiens chromosome 9 open reading frame 150 (C9orf150), mRNA.

Homo sapiens pellino homolog 2 (Drosophila) (PELI2), mRNA.

Homo sapiens tousled-like kinase 2 (TLK2), mRNA.

Homo sapiens chromosome 14 open reading frame 43 (C14orf43), mRNA.

Homo sapiens E2F transcription factor 3 (E2F3), mRNA.

Homo sapiens ELL associated factor 1 (EAF1), mRNA.

Homo sapiens bromodomain and PHD finger containing, 1 (BRPF1), transcript variant 1/2, mRNA.

Homo sapiens UV radiation resistance associated gene (UVRAG), mRNA.

Homo sapiens solute carrier family 7 (cationic amino acid transporter, y+ system), member 6 (SLC7A6), mRNA.

Homo sapiens protein phosphatase 1, catalytic subunit, alpha isoform (PPP1CA), transcript variant 1/2, mRNA.

Homo sapiens SWI/SNF related, matrix associated, actin dependent regulator of chromatin, subfamily d, member 2 (SMARCD2), mRNA.

Homo sapiens polyhomeotic-like 2 (Drosophila) (PHC2), transcript variant 1/2, mRNA.

Homo sapiens poly(A) polymerase alpha (PAPOLA), mRNA.
